# A description of malaria sentinel surveillance: a case study in Oromia Regional State, Ethiopia

**DOI:** 10.1186/1475-2875-13-88

**Published:** 2014-03-11

**Authors:** Joshua O Yukich, Jessica Butts, Melody Miles, Yemane Berhane, Honelgn Nahusenay, Joseph L Malone, Gunawardena Dissanayake, Richard Reithinger, Joseph Keating

**Affiliations:** 1 Center for Applied Malaria Research and Evaluation, Department of Global Health Systems and Development, Tulane University School of Public Health and Tropical Medicine, 1440 Canal Street, Suite 2200, New Orleans, LA 70112, USA; 2 U.S. President’s Malaria Initiative, Malaria Branch, U.S. Centers for Disease Control and Prevention, 1600 Clifton Road, Atlanta, Georgia 30333, USA; 3 Bill and Melinda Gates Foundation, 500 Fifth Avenue North, Seattle, WA 98102, USA; 4 Addis Continental Institute of Public Health, Addis Ababa, Ethiopia; 5 U.S. President’s Malaria Initiative, U.S. Centers for Disease Control and Prevention, Addis Ababa, Ethiopia; 6 U.S. President’s Malaria Initiative, U.S. Agency for International Development, Addis Ababa, Ethiopia; 7 RTI International, Washington, DC, USA

**Keywords:** Malaria, Epidemic detection, Short message system (SMS), Health facility, Sentinel surveillance, Ethiopia

## Abstract

**Background:**

In the context of the massive scale up of malaria interventions, there is increasing recognition that the current capacity of routine malaria surveillance conducted in most African countries through integrated health management information systems is inadequate. The timeliness of reporting to higher levels of the health system through health management information systems is often too slow for rapid action on focal infectious diseases such as malaria. The purpose of this paper is to: 1) describe the implementation of a malaria sentinel surveillance system in Ethiopia to help fill this gap; 2) describe data use for epidemic detection and response as well as programmatic decision making; and 3) discuss lessons learned in the context of creating and running this system.

**Case description:**

As part of a comprehensive strategy to monitor malaria trends in Oromia Regional State, Ethiopia, a system of ten malaria sentinel sites was established to collect data on key malaria morbidity and mortality indicators. To ensure the sentinel surveillance system provides timely, actionable data, the sentinel facilities send aggregate data weekly through short message service (SMS) to a central database server. Bland-Altman plots and Poisson regression models were used to investigate concordance of malaria indicator reports and malaria trends over time, respectively.

**Discussion:**

This paper describes three implementation challenges that impacted system performance in terms of: 1) ensuring a timely and accurate data reporting process; 2) capturing complete and accurate patient-level data; and 3) expanding the usefulness and generalizability of the system’s data to monitor progress towards the national malaria control goals of reducing malaria deaths and eventual elimination of transmission.

**Conclusions:**

The use of SMS for reporting surveillance data was identified as a promising practice for accurately tracking malaria trends in Oromia. The rapid spread of this technology across Africa offers promising opportunities to collect and disseminate surveillance data in a timely way. High quality malaria surveillance in Ethiopia remains a resource intensive activity and extending the generalizability of sentinel surveillance findings to other contexts remains a major limitation of these strategies.

## Background

Disease surveillance is one of the fundamental functions of public health systems [1], and recording and reporting of malaria cases is practiced in nearly all countries where transmission persists. Malaria remains a reportable disease even in countries, such as the United States of America, where sustained autochthonous transmission has long been essentially absent [2]. To realistically embark on the road towards malaria elimination, timely provision of accurate malaria surveillance data is necessary [3]. In the context of the massive scale up of malaria interventions, there is increasing recognition that the current capacity of routine malaria surveillance, conducted in most African countries through integrated health management information systems (HMIS), is inadequate: indicators are poorly defined; data reporting and completeness are often of unknown quality; and timeliness can be extremely variable. Further, there is often limited capacity for data analysis, data interpretation, and action. Additionally, in many locations confirmatory malaria diagnosis is of variable quality or absent [4,5].

In most sub-Saharan African (SSA) countries, routine malaria surveillance involves collecting and reporting aggregate data from public health facilities through the national HMIS. Data are collected about patients presenting at public facilities with symptoms of disease, and malaria cases are reported based on the diagnostic services available at the facility, or sometimes reported on clinical signs and symptoms only when diagnostic tools are absent. Where laboratory services are available, microscopy has generally been the most common form of confirmatory diagnosis. Although microscopy is generally considered the gold standard for malaria laboratory diagnosis, rapid diagnostic tests (RDT) have recently been rolled out in many SSA settings, particularly at the community level where microscopy is generally not available. This expansion may help to rationalize treatment practices and to improve the quality of surveillance data in many locations [6,7]. Data about malaria illnesses, with or without laboratory diagnostic confirmation, tend to be compiled weekly or monthly at each health facility and then reported up a vertical chain, with further aggregation at each level in the health system until reaching the most central level, such as the HMIS surveillance headquarters. These aggregated data are then ideally used by National Malaria Control Programmes (NMCP) and Ministries of Health (MoH) to make decisions related to the timing and frequency of prevention, treatment, and control interventions.

In general, routine surveillance systems tend to miss many malaria cases because they are either treated outside the formal public health system, not treated at all; underreporting and failure to capture testing and treatment data within the system can also lead to missed malaria cases. Concurrently, routine surveillance data may vastly overestimate the burden of malaria within the public health system due to under-utilization or lack of diagnostics, and a consequent reliance on clinical diagnosis. Additional challenges, such as poor data recording practices and lack of supervision, have an unknown effect on routine system data quality. In some settings, failure of facilities to completely report upwards in the reporting chain also results in aggregation of incomplete datasets and generalized under-reporting of malaria burden. Additionally, the timeliness of reporting to higher levels of the health system is often too slow for rapid action on focal infectious diseases, such as malaria. Validation of such data is nearly impossible in the absence of gold standard data sources to assess the system’s sensitivity and diagnostic quality control, which is often logistically difficult for microscopy and currently unavailable for RDTs. Furthermore, support and supervision for surveillance activities is often lacking within the HMIS. Small-scale sentinel surveillance with enhanced supervision and rapid reporting mechanisms are a viable alternative to relying solely on data collected through the country’s routine HMIS and may provide the best available gold standard for malaria surveillance and epidemic detection.

Ethiopia’s national malaria control strategic plan includes goals to eliminate malaria in low-transmission areas and achieve near zero deaths due to malaria by 2015. To monitor progress towards these goals, a system for capturing both local and regional transmission is essential. Particularly, there is a need for an effective surveillance system that can target focal areas of infection, increase capacity to identify transmission hot spots, and monitor near real-time malaria data to rapidly identify changes in malaria transmission, morbidity and mortality. The purpose of this paper is to: 1) describe the design and implementation of a malaria sentinel surveillance system in Oromia, Ethiopia; 2) describe data use for epidemic detection and response, as well as programmatic decision making; and 3) discuss lessons learned related to creating and running this system to provide practical examples and suggestions for improvement and use in other systems or settings.

## Case description

As part of a comprehensive strategy to monitor malaria trends in Oromia Regional State, Ethiopia, a system of ten malaria sentinel sites was initially established in early 2010 to collect data on key malaria morbidity and mortality indicators. This work was supported by the U.S. President’s Malaria Initiative (PMI) and implemented by Tulane University and Addis Continental Institute of Public Health in close collaboration with a range of stakeholders, including the Federal Ministry of Public Health (FMOH), Oromia Regional Health Bureau (ORHB), and the Ethiopian Health Nutrition and Research Institute (EHNRI). This work was approved by the Biomedical Institutional Review Board of Tulane University and the Ethical Review Committee of the Addis Continental Institute of Public Health.

The strengths and weaknesses of the system were assessed by a combination of methods over three years of implementation using various tools including: needs assessments, stakeholder interviews, supervision reports, data quality audits, laboratory and system quality control assessments, and analysis of surveillance data. These data formed the framework for assessing the lessons learned and best practices presented below. Methods for data quality audits and the general framework for assessment were based on frameworks outlined in U.S. Centres for Disease Control (CDC) guidelines [8,9].

### Malaria situation in Ethiopia

In Ethiopia, altitude and climate are the most important determinants of malaria transmission, which is highly seasonal and predominantly unstable [10]. The peak malaria transmission season occurs between September–December, while a minor transmission season occurs in April–May. There are four major eco-epidemiological malaria transmission strata in Ethiopia: 1) malaria-free highland areas above 2,500-meter altitude; 2) highland fringe areas between 1,500 and 2,500 meters (affected by frequent epidemics); 3) lowland areas below 1,500 metres (seasonal pattern of transmission); and 4) stable malaria areas (year-round transmission; limited to the western lowlands and river basins)[11]. *Plasmodium falciparum* and *Plasmodium vivax* are the dominant malaria parasites in Ethiopia, with their relative contribution thought to be approximately 60% and 40% of all malaria cases, respectively. *Plasmodium malariae* accounts for less than 1% of cases and *Plasmodium ovale* is very rare [12-14]. The primary malaria vector in Ethiopia is *Anopheles arabiensis*; secondary vectors include *Anopheles pharoensis*, *Anopheles funestus* and *Anopheles nili*[11,15-18]*.* Since little malaria transmission is apparent at altitudes above 2,000 metres, most malaria interventions, including insecticide-treated nets, are targeted to areas below 2,000 metres [11].

### Selection of sentinel surveillance sites

Ten primary health care units (PHCU) in Oromia were initially selected as sentinel sites for malaria surveillance activities. PHCU serve a catchment area of ~25,000 people and consist of district (*woreda*)-level health centres and satellite community (*kebele*)-level health posts. Health centres are primarily concentrated in urban areas, and are usually staffed by at least one health officer, laboratory technicians, pharmacists or druggists, and midwives. Most health centres have inpatient capabilities, albeit limited, and as such they are the first referral point for severe malaria cases identified at health posts. Health posts are usually in rural areas and focus on preventive services, providing only limited curative services, mainly for malaria. Health posts are staffed by two health extension workers (HEWs), who are fully-salaried FMOH staff. These HEWs are primarily women with a high school diploma and typically originate in the communities they serve. HEWs provide service delivery on 16 selected health packages for which they receive specific training, including malaria. They are expected to confirm all suspected malaria cases (fever cases) with a multi-species RDT and provide appropriate treatment based on RDT results.

**Figure 1:**
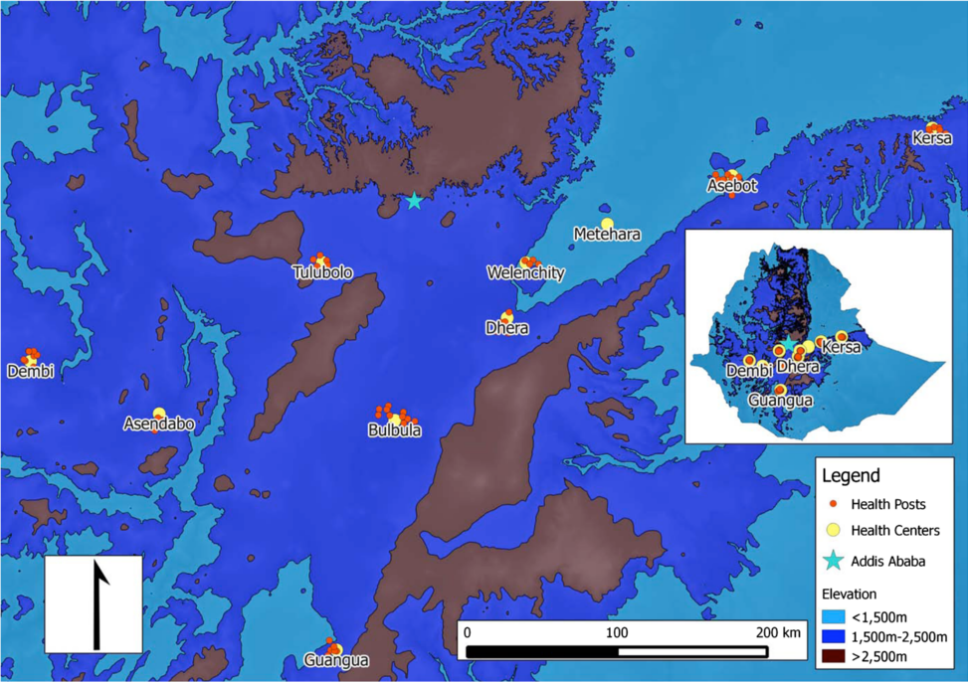
Map of sentinel facilities.

Following a national stakeholder meeting, the following criteria were used for selecting PHCUs, to serve as sentinel sites: 1) presence of an outpatient clinic that sees an average of at least 50 patients per day; 2) laboratory capacity to diagnose malaria using microscopy; 3) ability to provide artemisinin combination therapy (ACT) as first-line treatment for uncomplicated malaria during selection visit; 4) pre-existing designated personnel responsible for data collection and reporting at the facility during selection visit; 5) situated below 2,000 metres above sea level in a malaria transmission area; and 6) available electricity and year-round access via road. A needs assessment was conducted at 20 health centres which met the above criteria prior to final consideration and selection. Health centres meeting the above criteria were considered by the ORHB; community acceptance and political commitment were other important final criteria for selection. Efforts were also made to include health centres in areas where the timing, duration, and intensity of transmission varied (e.g. more than 6 months transmission and less than three months transmission; less than one confirmed case/500 persons per year and more than one confirmed case/500 persons per year; predominance of *P. falciparum* or *P. vivax*), and where both microscopy and RDTs were used. Health centres formed the initial and central points for each sentinel surveillance site; over time expansion to all satellite health posts within the 10 PHCUs was completed. Full expansion of the malaria sentinel surveillance system was achieved in 2012 (after approximately 2 years), it included 10 health centres and their 73 satellite health posts (Figure 1).

### Data collection and reporting

Patient data were collected at two main service delivery points: health centres and health posts. At health centres, an outpatient department (OPD) register and a laboratory register captured patient demographic and malaria data. The OPD registers were designed to capture information on patient age, location of residence, fever history, laboratory tests requested, laboratory results for malaria and relapsing fever through microscopic examination of Giemsa or Field stained blood slides or multi-species RDT (CareStart®, AccessBio, NJ), species-specific final diagnosis (i.e., uncomplicated malaria, severe [complicated] malaria, other), drugs prescribed, inpatient admittance, death and referral to higher level facilities. At health posts, data were collected from the routine fever and malaria patient register. Because HEWs at health posts can currently only provide treatment for one acute illness (malaria infection), one patient register covers all diagnostic testing and treatment services.

Surveillance field support staff initially visited each health centre every two weeks (after one year this was changed to monthly) to work with health centre staff to extract relevant malaria data from the registries and share findings from previous months and from other facilities in the surveillance network. Data in the field were collected using paper forms; after each visit the field support staff entered all data into a pre-populated data base IV format (dbfIV) file using EpiInfo 7.0 (U.S. Centers for Disease Control and Prevention). Additional points of communication and data collection at health facilities included the medical stores and dispensaries, where stocks of malaria-related commodities are tracked. Health posts were visited monthly to extract relevant malaria data from paper registers. In addition to register abstraction, all health posts and health centres sent data electronically in aggregate on a weekly basis via short message systems (SMS). Data from the SMS system are maintained in a separate database, as paper registry and laboratory records were considered gold standard data. These data were compared using methods discussed below to ensure accuracy of rapid reporting data.

**Figure 2:**
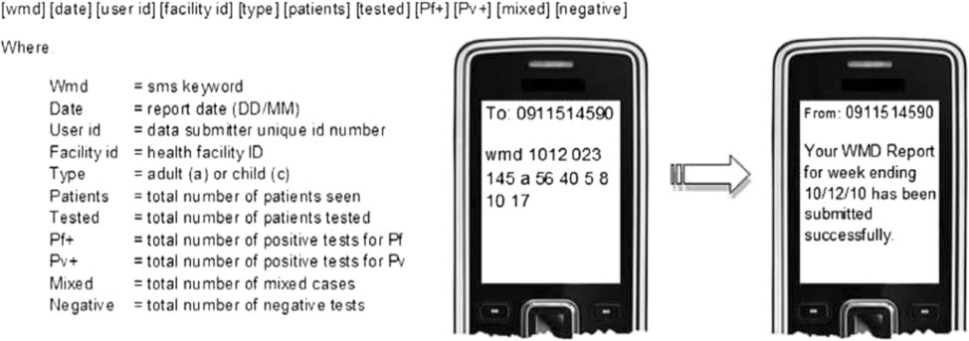
Screen shot of mobile phone displaying data reporting format used in Ethiopia at the health post level.

To ensure the sentinel surveillance system provides timely, actionable data, the sentinel facilities send weekly SMS data messages to a central server. These messages provide essential malaria data to notify health officials of potential case build-ups requiring a targeted response; the paper data provide more detailed data for in-depth analysis. The main system components of the SMS-based data collection are a multi-channel communication processor (MMP) and a management and reporting portal (MRP). The MMP is the component used to manage channel communications, such as SMS. The MRP is browser-based and interfaces with various desktop applications such as Microsoft Excel or Adobe Reader to allow for reports to be exported in various formats. The entire system interfaces with the Ethiopian Telecommunications Corporation to facilitate communication via SMS and the internet. There are three general system users of the data: 1) data submitters (e.g., HEW at health posts and laboratory technicians at health centres); 2) data users (e.g., stakeholders, data managers at project institutions, health workers); and 3) administrators (e.g., field support team members at the implementing partner institution). Figure 2 presents a mobile phone screen shot to illustrate the data reporting format; it also lists the indicators captured as part of the systems’ weekly malaria reports. All data in monthly reports are presented in tabular and graphic formats.

The web-based interface allows users to view the data in near real-time for assessment of expected and actual malaria cases occurring within the defined area. A second mechanism of epidemic notification occurs in the form of both an email and SMS text sent to the users when a specific threshold is reached. For example, when a data report is submitted, the system automatically checks the values of *P. falciparum* and *P. vivax* test positivity rates (TPR) as well as numbers of laboratory-confirmed cases against predefined threshold values for that health facility. If any of the indicators meet or exceed the threshold value for that facility in a given week, the system automatically sends an e-mail and text to the health centre or post, or to other designated users where the epidemic notification had been triggered. The message includes the indicator that triggered the alert, the current data value, the threshold value, and the catchment area to which the facility is associated.

The system interacts with the standard surveillance system for malaria in Ethiopia at multiple points. These include notification of relevant officials at all levels, including woreda malaria focal persons, of unusual malaria morbidity or mortality reports, in-person and telephone follow-up at the woreda and facility level for routine supervision and the communication of results of surveillance activities to the higher levels of the Ethiopian health system including the Federal Ministry of Health.

### Data quality assurance

To reduce the potential for SMS system error, several automatic basic data logic checks are employed on reports. These include checks to ensure that confirmed cases do not exceed malaria tests conducted and other similar logical checks. If an SMS data report fails to meet these checks, the surveillance system sends a message to the sender indicating a resubmission is necessary. The system overwrites the original report once a resubmission has been submitted, and a message confirming receipt of the new report is automatically sent to the sender. To ensure data quality on paper forms, every month members of the field support team visit each health centre to check registers against physician and patient records. All data from OPD and laboratory registers are recorded in triplicate during the patient encounter. During field support visits, members of the team conducting supervision extract one copy of the register pages; using a copy of the source data instead of transcribing the source data removes one level of possible error. Where register data are incomplete, reconciliation is attempted using the routine physician patient records (*i.e.,* OPD cards). Records are also cross-checked with the routine laboratory and dispensary registers to confirm tests were performed and drugs dispensed, as indicated.

**Figure 3:**
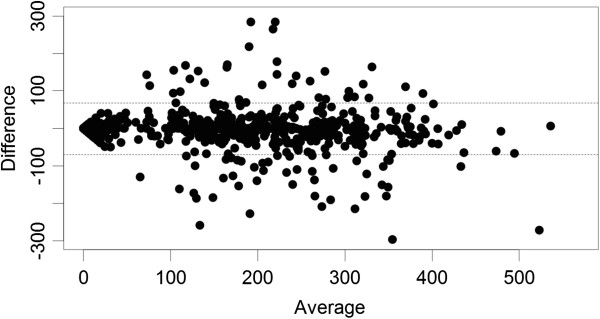
**Bland-Altman plot of total outpatients seen at each facility (health centres and health posts) per week from the SMS system and the paper system during 2011.** The difference between the two measures is shown on the y-axis and the average of the two measures is shown on the x-axis.

**Figure 4:**
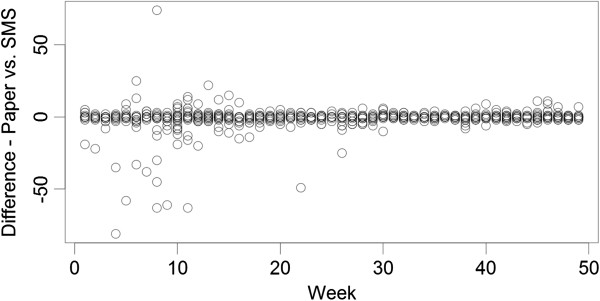
**Plot of differences between paper and SMS reports of confirmed *Plasmodium falciparum *** **cases per week over time at all facilities (health centres and health posts).**

At the team headquarters in Addis Ababa, all OPD and laboratory data are entered in duplicate into a .dbf IV file format to create an individual patient-level dataset for each facility. Within the SMS system, data are submitted electronically and automatically entered into a seperate database. An external assessment of this system was conducted in April 2011, one year after implementation (data not shown). To verify quality of data collected through SMS reports in an internal audit, weekly aggregate indicators were re-calculated using the primary paper registry data from all 83 sites for 2011 and compared to results from the SMS weekly aggregate reports, yielding 2,759 weekly reports from the SMS dataset and 2,759 corresponding observations based on paper registries. The paper data consisting of individual patient records were used to re-calculate all weekly indicators by project staff and compared to the corresponding SMS reports at health centres and health posts using a Bland-Altman framework [19,20], wherein the difference between two measurements (or reports) is plotted against the average of the two measurements so that both magnitude of error as well as trends in error with measurement size (bias), can be visualized and quantified. Measurement differences were also plotted by time. While this analysis was conducted for all indicators presented here are results on the number of confirmed *P. falciparum* cases reported only, as this is generally felt to be the most sensitive and important public health indicator collected by the system. The results indicate that overall concordance between the two reporting systems (paper recording and SMS reports) is high (Spearman’s ρ was greater than 0.73 for all indicators with only one result showing a ρ less than 0.79), but that the size of error was likely to grow with the size of the actual weekly indicator reported, at least at very low ranges of the average of the indicators calculated using the SMS and the paper registry data (Figure 3). Only 80 facility reports of the 2,759 week reports (including health centres and health posts) (2.9%) showed a difference between SMS and paper reports of more than ±75 patients per week. A large fraction of these cases were due to missing SMS reports in high volume weeks based on the paper records. The concordance of SMS reporting with paper records appeared to improve after a relatively short 15-week “burn-in” period (Figure 4). When normalized to the average of the two reports, differences between the two reporting systems were relatively larger when reporting small numbers for a specific indicator, but these fell to very low relative error sizes when reporting larger numbers.

**Figure 5:**
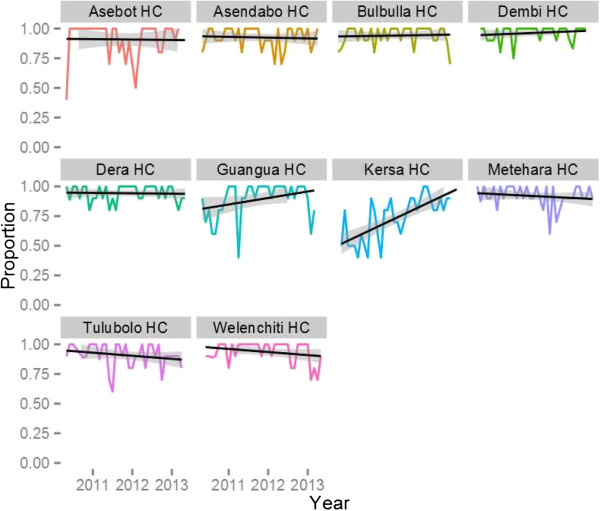
Results of Laboratory External Quality Assurance for malaria microscopy: proportion of blood slides read correctly for presence of malaria parasites at health centres (black lines are linear trend lines).

An External Quality Assurance (EQA) system for malaria microscopy was developed at the start of the project based on WHO guidelines to ensure that quality diagnostics were being applied at the health centre level (i.e. where microscopy remains the standard method for laboratory confirmation of malaria infection). The system sampled ten slides per month (stratified to include five positive slide and five negative slides using systematic random sampling from the laboratory register). Slides were re-read by an expert microscopist blinded to the facility result and re-read by a second expert microscopist centrally in the event that the gold standard reading was in disagreement with the facility result. Gold standard results were considered to be the two readings which were in agreement. Analysis of EQA data was reported back to facilities during monthly meetings combined with advice about the proper preparation and reading of slides based on an expert review of slide staining technique. Results of the EQA are presented in Figure 5. Overall concordance on both positivity and species identification were relatively high with >75% average for each facility, but imperfect with significant month to month variation. Logistic random effects regression analysis showed that overall there was no significant trend in accuracy for malaria diagnosis over time across all ten health centres, but that performance varied significantly by facility; within facility trends only appeared statistically significant in two facilities (both were positive). One facility performed especially poorly initially, but also showed the largest improvement over time.

### Malaria at sentinel sites

Data on outpatient testing and laboratory confirmation of malaria cases has been collected in the sentinel sites over a period of 43 months. To examine trends in cases over time, multilevel time series Poisson random effects regression models were fit to data across all sites and over the entire time series with fixed effect covariates for month and year (either as a factor variable or as a linear time trend). Outcome variables were either all confirmed malaria cases or species-specific confirmed malaria cases (*P. falciparum* or *P. vivax*) with the exposure of total outpatients attending the facility during the month. Random intercepts were included for both the facility and PHCUs (i.e. health centres/satellite health posts).

**Figure 6:**
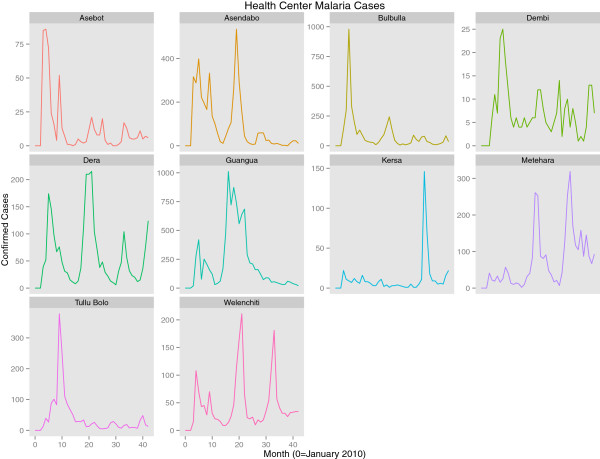
Trends in confirmed malaria cases at all sentinel health centres from January 2010 to August, 2013.

**Figure 7:**
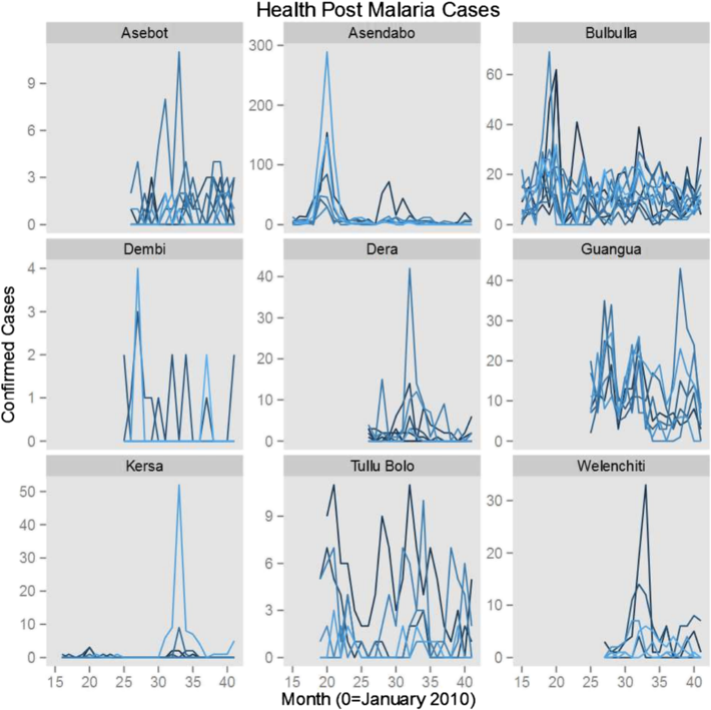
Trends in confirmed malaria cases at all sentinel health posts (grouped by their reporting health centre).

Data indicate a downward trend in overall confirmed malaria cases across sentinel sites between 2010 and 2013, after adjusting for seasonality and the variation in incidence levels between individual facilities and across PHCU (Table 1). They also indicate that the trend was much more prominent in *P. vivax* than in *P. falciparum* cases. Linear trend models show approximately a 13% (95% CI 11.2 – 14.8) reduction per year of *P. falciparum* cases; treating year as a factor suggests that most of this decline resulted from a 27% reduction in 2012 compared to 2010 levels. Early data from 2013 indicate a return in *P. falciparum* case levels similar to that observed in 2010. These results indicate that changes in *P. falciparum* cases at the sentinel sites most likely represent inter-annual variation rather than a general trend. Model results for *P. falciparum* alone are only presented with a linear trend for consistency across the three outcomes (*P. falciparum*, *P. vivax* and all malaria cases) but are best interpreted using the year factor model. These models do not adjust for rainfall or other possible confounders and, thus, make no effort to explain the reasons behind trends, but only seek to accurately estimate the overall trend in malaria incidence in the sentinel facilities. While full data for 2013 were not available at the time of manuscript submission, the inclusion of month in the models to account for seasonality provides the ability to estimate whether 2013 malaria incidence was higher or lower for the months with already available data. Overall the incidence rate ratios indicate that across the sentinel sites there has been an approximately 15% (95% CI 13.8%-16.2%) fall in overall incidence rates per year since 2010 (Table 1, Figures 6 and 7). By June 2013, the sentinel surveillance system had identified three major malaria epidemics: a *P. falciparum* epidemic occurring in the catchment of Bulbulla Health Centre in June 2010; a *P. vivax* epidemic occurring in the catchment of Tulu Bollo Health Centre in October 2010; and a mixed but largely *P. falciparum* epidemic occurring in the catchment of Guangua Health Centre which lasted through the majority of 2011. Several smaller malaria case buildups were also noted by the system as well as several small outbreaks of relapsing fever.

**Table 1:** Regression modeled estimates of trends in malaria cases at all sentinel sites

**Model**	**N (facility-month observations)**	**Coefficient**	**Incidence rate ratio**	**Standard error**	**p-value**
**All cases linear trend**	1528	Year (Linear trend)	0.85	.006	<0.001
**All cases year factor**	1528	Year 2010	Ref	-	-
		Year 2011	1.04	.014	0.010
		Year 2012	.71	.011	<0.001
		Year 2013	.78	.028	<0.001
***P. falciparum *** **cases linear trend**	1528	Year	.87	.009	<0.001
***P. falciparum *** **cases year factor**	1528	Year 2010	Ref	-	-
		Year 2011	1.19	.024	<0.001
		Year 2012	.74	.017	<0.001
		Year 2013	.99	.065	0.860
***P. vivax *** **cases linear trend**	1528	Year (Linear trend)	.80	.008	<0.001
***P. vivax *** **cases year factor**	1528	Year 2010	Ref	-	-
		Year 2011	.85	.017	<0.001
		Year 2012	.65	.014	<0.001
		Year 2013	.46	.023	<0.001

All regression models include month of the year as a factor variable, and random intercepts at both the primary health care unit level and the health facility level.

**Figure 8:**
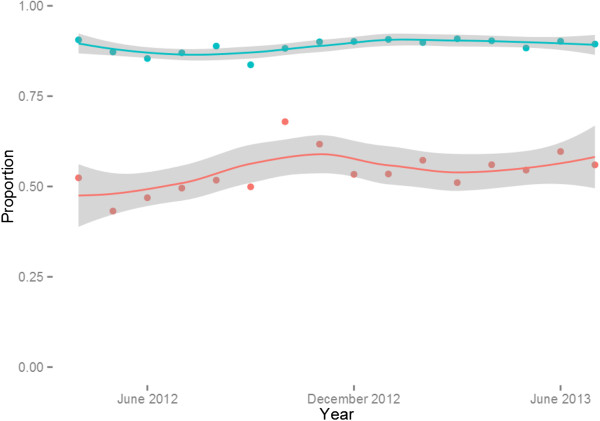
**Proportion of patients and confirmed malaria cases seen at health centres (as compared to Health Posts) after full scale up of surveillance at Health Post level.** (blue line reflects all OPD patients and red line reflects all confirmed cases).

Figure 8 presents the proportion of laboratory cases and total outpatients seen at the health centre and health post level after full scale up of surveillance to all health posts. The results indicate that the health centres see the vast majority of all patients, typically greater than 80% in any given month without much evidence of seasonal trends, while approximately half of the malaria burden based on confirmed cases is seen at the community level. As no control data is available it cannot be determined from these data whether this reflects a shift in treatment seeking from health centres to the community level after the development of the Health Extension Program, which resulted in the mass deployment of HEWs at the community level in Ethiopia, or an overall increase in treatment-seeking. Data from two malaria indicator surveys (2007 and 2011), however, documented a dramatic increase in the proportion of febrile children seeking treatment within 24 hours (15% to 51%) between the two surveys (MIS 2007 and 2011) [13,21]. During the same period (2007–2011) the number of health posts in Ethiopia increased by more than 150%, indicating that the burden treated at the community (health post) level may indeed have been largely missed in surveillance systems prior to the availability of community level treatment and surveillance. Only two malaria specific deaths were recorded in the health facilities during the period of observation.

## Discussion

This sentinel surveillance system captures data related to malaria morbidity, mortality, diagnosis, and treatment. The discussion of this paper focuses on three key implementation challenges affecting data interpretation, as well as possible solutions. The three challenges include: the data recording and reporting process, use of diagnostics, and data use for decision-making.

### Generating timely and accurate data

Although the paper-based data reporting process at sentinel sites has been successful in delivering quality data from health centres and health posts to stakeholders on a monthly basis, the reliance on periodic field-based support visits for data collection slowed the process and was resource-intensive. Additionally, rapid reporting became even more challenging when health post (community level) data were included. The integration of weekly SMS reporting greatly improved the timeliness of the surveillance system with relatively little loss of fidelity, especially after a “burn-in” period. Use of SMS and overall maturation of the system allowed the scaling back of supervision from twice monthly to once monthly, thus improving the overall efficiency of the surveillance system. Over time, supervision could potentially be reduced to once every two or three months, assuming staff turnover at health facilities is kept to a minimum.

### Capturing complete and accurate patient level data

The sentinel surveillance system protocol requires that clinicians and laboratory staff enter data in their respective registers at the time of the patient encounter (or accurately capture such data from patient cards at a later date). Laboratory registers in Ethiopia have remained consistent, reliably filled and comprehensive. However, OPD registers have at times been incomplete, particularly for identifying suspect malaria cases; a problem which will not be remedied by the use of SMS reporting systems. The OPD registers’ incompleteness is further compounded by the use of multiple data forms, including an HMIS register that does not capture the necessary data for malaria surveillance (*e.g.* HMIS indicators currently capture neither testing rates nor consistently differentiate between laboratory confirmed and clinical malaria diagnoses). Failure to fully complete OPD registers used by the surveillance sites may be complicated by a high reporting burden at the facility level for both malaria and non-malaria illnesses. This burden may include in some locations newly expanded HMIS clinic registers, integrated disease surveillance and response (IDSR) reports and other HMIS and surveillance reports. Incomplete OPD registers may influence measures of appropriate testing of suspect malaria cases; records of a patient’s fever history or clinical suspicion of malaria are required to calculate testing rates, a key performance indicator for facilities scaling up universal diagnosis for suspect cases. Improved patient cards which integrate data collection needs of the HMIS with malaria surveillance data needs could possibly be used to remedy this problem. Furthermore, the role of the district health office (or equivalent) in the system should be reinforced by establishing a focal point at the district office to serve as the liaison between surveillance systems and the health facilities.

Challenges related to incomplete data at health facilities highlight the importance of collaboration and coordinating data collection across multiple branches of the health system. If the HMIS captured all of the key indicators needed to inform malaria control, parallel data collection systems for malaria surveillance might not be necessary. Clearly, an extra layer of enhanced malaria indicator data collection and reporting does potentially create a reporting burden: staff may be resistant to the additional reporting, and may resist participation in the data collection and reporting process, which in turn may contribute to reduced data quality and potentially make measurement of certain trends unreliable. Towards the end of the global malaria eradication efforts in Ethiopia, diagnosis and treatment for malaria was re-integrated into the overall health system and multiple reporting mechanisms existed for disease surveillance. Over time these came to include the HMIS, IDSR, public health emergency management (PHEM) and sentinel surveillance systems. Failure to harmonize data collection and indicators across systems leads to increased reporting burdens, and conflicting estimates of indicators [14].

In most locations, government-provided public health care is only one of the many possible options that patients use to seek care for febrile illness, and malaria transmission and treatment-seeking patterns may fluctuate over time [22-24]. Given these issues, surveillance systems must be able to account for changes in patient treatment-seeking behaviour and provider practices, and should expand the points of data collection from one type of public health facility to an assortment of providers, including multiple health system levels in the same geographic areas, as well as the potential incorporation of private providers if monitoring trends in morbidity and mortality are a focus of the system.

The malaria diagnostic protocol for the sentinel surveillance system requires laboratory confirmation of all suspect cases by either RDT or microscopy. Adherence to the diagnostic protocol was measured by calculating test uptake from the OPD and laboratory registers: the number of patients tested (from laboratory register) divided by the number of patients with suspect malaria (from the OPD register). The number of cases tested in some facility-month reports were far greater than the number of suspect cases registered through the OPD, resulting in reported test uptake over 100%, and suggesting the possibility that some patients are receiving laboratory services for malaria without first registering, seeing a clinician, and being recorded in the reviewed OPD registers as a suspect case. Alternative explanations for this discrepancy include: incomplete registration of patients in OPD registers, referrals to the laboratory from service departments at health centres other than the OPD, and the potential for clinicians who use the malaria diagnostic test to rule out infection to refer patients for testing who they do not consider as having suspected malaria. Unfortunately, matching data from OPD registers to laboratory registers has been challenging due to the frequent duplication of patient OPD card numbers. The challenges associated with calculating test uptake using two separate and discrepant data sources makes this indicator of little use to the system. This indicator can be calculated only using OPD records, but the implications in terms of bias of doing so are currently not known.

### Making data useful and generalizable

One primary purpose of this sentinel surveillance system was to detect malaria outbreaks through regular analysis and reporting of data to inform appropriate public health responses. By June 2013, the sentinel surveillance system had identified three major malaria epidemics: a *P. falciparum* epidemic at Bulbula Health Centre in June 2010, a *P. vivax* epidemic at Tulu Bollo Health Centre in October 2010, and a mixed but largely *P. falciparum* epidemic at Guangua Health Centre which lasted through the majority of 2011. Standard responses to these epidemics were to notify local health authorities and malaria control partners, and to ensure that health centres and health posts were adequately supplied for testing and treatment; local partners and authorities also increased targeted communication interventions in the area, and in some cases targeted indoor residual spraying of households with insecticide was also used (in the Guangua epidemic).

There are inherent challenges in applying standard epidemic detection methods depending upon the country context. In the absence of high quality historical data on malaria incidence, application of methods such as the WHO monthly methods or standard process control techniques is challenging or inappropriate. For this reason the project has moved toward the use of spatio-temporal regression methods for detection of increased local incidence of malaria. Collecting information on place of residence may aid in the identification of spatial clustering of cases; however, the challenges in reconciling local place names when literacy levels in the population are low, standardized maps and place names do not exist, and discordant place names appear in multiple registers for the same patient should not be underestimated.

Reliance on historical data or malaria microscopy may also be a poor indicator of malaria clinical incidence. At the health post level, multi-species malaria RDTs are utilized exclusively, and it currently appears that at least half of all malaria cases in Ethiopia are diagnosed based upon RDT results. Ethiopia has low malaria transmission levels as evidenced by the 1.3% microscopy slide prevalence found in the Malaria Indicator Survey performed in 2011 [13,21]. When there is very low malaria transmission, false negativity due to low sensitivity of both RDTs and microscopy at low parasite densities may prove problematic [25-27]. Conversely, false positives may lead to overuse of anti-malarial drugs and a failure to properly delineate areas of the country absent of malaria risk. Malaria prevention and control programmes must ensure quality of laboratory confirmation especially in low transmission areas.

Data from the sentinel surveillance system are also used for informing routine program decision making, such as monitoring commodity stocks and forecasting demand, estimating and forecasting the burden of malaria expected at health posts, health centres and hospitals. During support visits, staff review data and notify the district office and the health centre director of any substantial increase in malaria cases. Monthly summary reports from all ten sentinel districts are also shared with senior ORHB officials, senior PHEM officials and with the FMOH for their information and action. On several occasions reports of malaria case buildups from districts nearby the sentinel sites prompted queries from the FMOH as to whether similar trends could be confirmed at nearby sentinel sites. Additionally, these officials were able to check case summaries in real-time by health facility and health post as soon as they were posted online by weekly SMS malaria morbidity reports.

While sentinel surveillance systems often lack the ability to produce generalizable data beyond catchment areas, they can provide precise, accurate and real-time data from discrete areas that can assist in interpreting events that are in nearby districts and aggregated data from other districts. Such systems could be testing grounds for operational strategies to identify foci of increased malaria transmission or to pilot enhanced control and elimination efforts, and provide an example of how to achieve high quality surveillance data in a resource limited setting. Ethiopia’s sentinel sites are currently serving as grounds for testing strategies to explicitly incorporate geo-spatial information on cases and travel histories into routinely collected facility-based data, as well as piloting the use of spatio-temporal regression analyses to identify locations with increased malaria risk to enhance the sensitivity and specificity of routine automated epidemic detection.

### Conclusions and recommendations

This paper describes three implementation challenges that impacted a malaria surveillance system’s performance: 1) ensuring a timely and accurate data reporting process; 2) capturing complete and accurate patient-level data; and 3) expanding the usefulness and generalizability of the system’s data to monitor progress towards the national malaria control goals of reducing malaria deaths and eventual elimination of transmission.

Transmission of data more rapidly and accurately, and expanded dissemination of malaria surveillance data can be achieved using mobile phone technology. Such data can accurately represent registry data in the aggregate, at least in Oromia, and allows for tracking of trends in malaria in surveillance districts. The increased use of this technology across Africa offers promising opportunities to collect and disseminate surveillance data in a timely way. High quality malaria surveillance in Ethiopia remains a resource intensive activity and extending the generalizability of sentinel surveillance findings to other contexts remains a major limitation of these strategies. Future sentinel surveillance activities should continue to focus on expanding and ensuring quality control for and widespread use of diagnostics, delivering rapid data transmission with mobile technology, and enhancing generalizability and representativeness through wide ranging and appropriate site selection and inclusion of community level testing and treatment in sentinel sites.

## Competing interests

JM, RR and GD worked for the funder of this research during the implementation of this project. No other authors have any competing interests.

## Authors’ contributions

JY and JK conceived of this study, collected data and drafted the manuscript. JM, RR and GD contributed to the development and implementation of the project, provided advice on the implementation and provided critical review of draft manuscripts. JB and MM contributed to the collection of data, and critically reviewed draft manuscripts. HN and YB contributed to the conception of the study, collected data, supported the analysis of data and critically reviewed draft manuscripts. All authors read and approved the final manuscript.
